# Diabetes and two kinds of primary tumors in a patient with thalassemia: a case report and literature review

**DOI:** 10.3389/fonc.2023.1207336

**Published:** 2023-08-11

**Authors:** Xiaoyan Yu, Yi Peng, Tingting Nie, Wenjia Sun, Yajuan Zhou

**Affiliations:** ^1^ Department of Radiotherapy, Hubei Cancer Hospital, Tongji Medical College, Huazhong University of Science and Technology, Wuhan, Hubei, China; ^2^ Department of Pathology, Hubei Cancer Hospital, Tongji Medical College, Huazhong University of Science and Technology, Wuhan, Hubei, China

**Keywords:** thalassemia, diabetes, nasopharyngeal carcinoma, tongue squamous cell carcinoma, iron overload (IOL), case report

## Abstract

**Background:**

Thalassemia is a group of common genetic hematologic disorders characterized by deficient synthesis of the hemoglobin chain. Due to effective blood transfusion and optimization of chelate therapy, the survival of thalassemia patients and their overall quality of life have improved noticeably in the past few decades. As a consequence, the longer life expectancy has led to the manifestation of several concomitant morbidities, including heart disease, infections, cirrhosis, endocrine abnormalities, various malignancies, and so on. In this context, the probability and updated literature about some malignancy cases in patients with thalassemia build new scenarios for the next few years. We describe the first report of a thalassemic patient developing diabetes and head and neck cancer and try to summarize the possible predisposing factors and mechanisms behind their phenomenon.

**Case presentation:**

The current case report describes a 50-year-old Asian man who has been diagnosed with thalassemia since childhood. In early 2017, he was also diagnosed with diabetes and started on insulin-hypoglycemic treatment. The patient was then diagnosed with primary non-keratinizing undifferentiated carcinoma of the nasopharynx in late February 2013. A biopsy of the left tongue revealed squamous cell carcinoma (SCC) in late March 2019.

**Conclusions:**

We report the first case of a thalassemic patient developing diabetes and squamous cell carcinoma of the head and neck and discuss the possibility of a link between the three diseases. This specific case should alert physicians to the possibility of endocrinopathy and malignancy in thalassemic patients.

## Introduction

Thalassemia is one of the most common inherited monogenic diseases in the world. It is estimated that nearly 1–5% of the global population are carriers of a thalassemia genetic mutation (1). Thalassemia is known to be highly prevalent in a zone that extends from the Mediterranean region through sub-Saharan Africa and the Middle East to the Indian subcontinent and East and Southeast Asia (2). However, because of increasing and continuing immigration, thalassemia is becoming a global disease burden (3). Thalassemia can be categorized into two types according to the patient’s clinical need for transfusion: transfusion-dependent thalassemia (TDT), which includes Bart’s hemoglobin, severe forms of HbE/β-thalassemia, and β-thalassemia major; and non-transfusion-dependent thalassemia (NTDT), which includes individuals with hemoglobin H disease, β-thalassemia intermedia, and mild to moderate forms of HbE/β-thalassemia (4). The thalassemia syndromes are a remarkably heterogeneous group of disorders with a broad spectrum of disease severity, characterized by varying degrees of chronic hemolysis, ineffective erythropoiesis, compensatory hemopoietic expansion, chronic hemolytic anemia, hypercoagulability, iron overload, and even more complex clinical phenotypes (5).

With more optimal therapeutic approaches and improvements in iron chelation treatment with deferoxamine, patients with thalassemia now have a better prognosis than before (6). At the same time, however, these patients are likely to continue to be affected by a large number of complications. A study by Borgna-Pignatti et al. showed that the most common complications leading to death in thalassemia patients were heart disease, infection, cirrhosis, thrombosis, malignancy, and diabetes (7). A national vertical cohort study was conducted by Chung et al., which showed that patients with thalassemia were more likely to have diabetes, and the overall risk of developing cancer in those patients was 1.47 times higher than in the comparison cohort after adjusting for age, gender, and comorbidities (8). There are a few articles about the coexistence of thalassemia and malignancies worldwide. However, until now, no head and neck tumor has ever been reported in this population. Here, we describe a case report of a thalassemia patient with diabetes and two types of primary head and neck tumors and further discuss its potential mechanism.

## Case presentation

**Figure 1:**
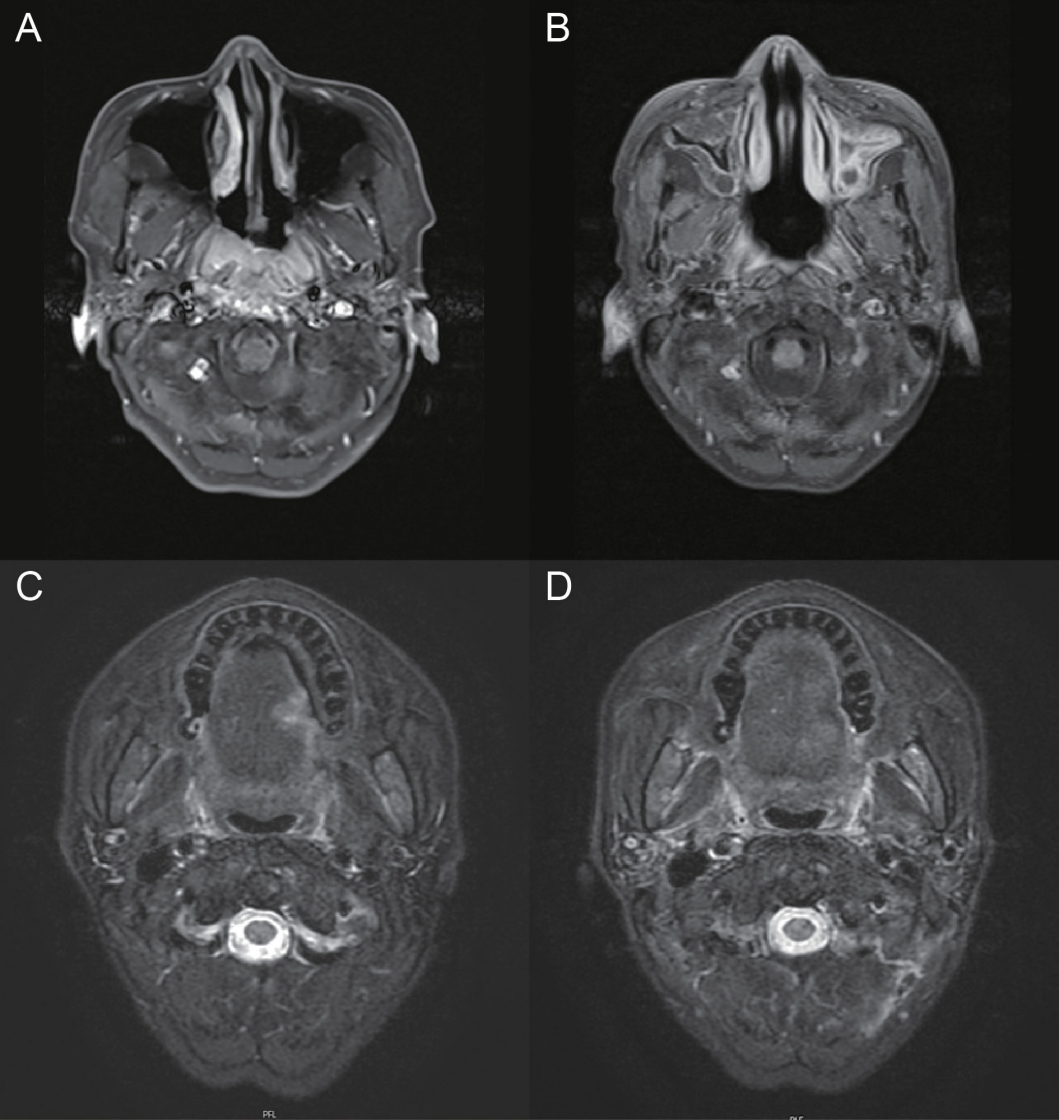
Pre- and post-treatment MRI of nasopharyngeal carcinoma and tongue cancer. **(A)** MRI depicting the nasopharyngeal lesion. **(B)** MRI showing the response of nasopharyngeal carcinoma after treatment. **(C)** MRI depicting the left tongue lesion. **(D)** MRI showing the response of tongue cancer after radiotherapy.

**Figure 2:**
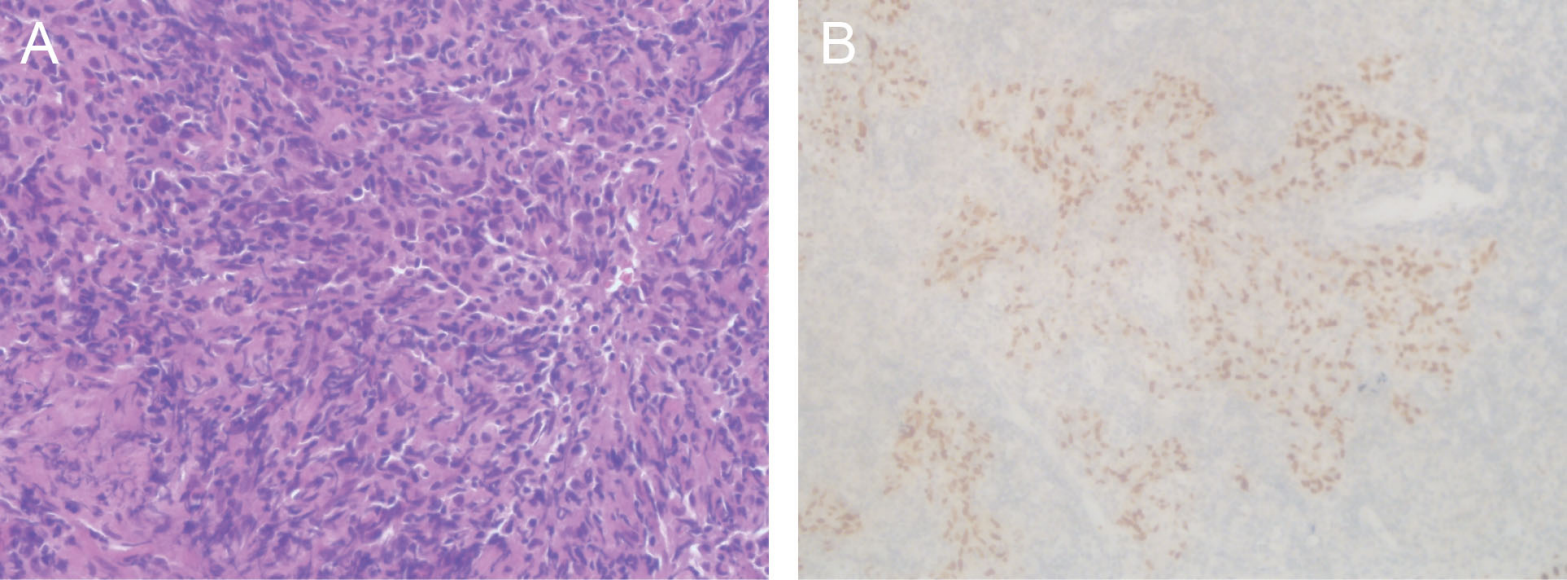
Histological images of the nasopharyngeal lesion. **(A)** Hematoxylin and eosin staining of primary nasopharyngectomy specimens demonstrated non-keratinizing undifferentiated carcinoma of the nasopharynx. **(B)** Immunohistochemical staining showed negative staining for p63. Magnifications: **(A)** ×400; **(B)** ×200.

The patient was a 50-year-old man with no history of smoking or alcohol consumption. He had been diagnosed with NTDT since childhood. In 1982, the patient underwent a splenectomy and was routinely given oral aspirin and hydroxyurea after surgery. After that, he was followed up many times and treated with blood transfusions at other sites due to severe hemoglobin decline. The hemogram showed abnormal proliferation of leukocytes and platelets and decreased hemoglobin. In early 2017, he was diagnosed with diabetes and then started on insulin-hypoglycemic treatment. In January 2013, he presented with discomfort in his right ear (aural fullness). The patient was diagnosed with primary non-keratinizing undifferentiated carcinoma of the nasopharynx (cT3N2M0 stage III, p63 positive) based on histopathologic and imaging findings conducted in late February of that same year (**Figures 1A**, **2**). The patient received two cycles of TPF (paclitaxel, cisplatin, and fluorouracil) systemic induction chemotherapy combined with radical intensity-modulated radiation therapy and two cycles of TPF adjuvant chemotherapy. On the multiple follow-up magnetic resonance imaging (MRI) scans after the completion of systemic therapy, the primary lesion of the nasopharynx and metastatic lymph nodes of the neck were continuously reduced. All malignant lesions resolved on 6 March 2014 (**Figure 1B**). At the beginning of 2018, the patient presented with an ulcer on the left side of the edge of the tongue with concurrent pain when eating and speaking, the size of which progressively increased over time. A biopsy of the left tongue revealed squamous cell carcinoma (SCC) with cT2N0M0 stage II at the end of March 2019 (**Figure 1C**). Considering the thalassemia, the patient and his family refused surgical treatment, so he was treated with three-dimensional conformal radiation therapy with a dose of 65Gy in 28 daily fractions from 16 April 2019 to 24 May 2019. MRI scans after radiotherapy showed that the lesion on the left edge of the tongue was smaller than before radiotherapy (**Figure 1D**). The wall of the nasopharynx, which was slightly thicker than before, showed no obvious change. There were many small lymph nodes in the IB area of the right neck, some of which were smaller than before. Considering the residual tumor and the fact that the patient didn't receive concurrent chemotherapy during radiotherapy, the patient received TS-1 maintenance chemotherapy on 13 June 2019 and was in regular follow-up in our department.

## Discussion

With more patients living longer, abnormalities in glucose homeostasis and solid and hematological malignancies are becoming increasingly pronounced. The actual mechanism of malignancies and diabetes development in thalassemia is unclear, but many possible hypotheses may be valid and meaningful.

Diabetes mellitus is an important endocrine disease resulting from transfusional hemosiderosis, which is found in 20–30% of thalassemia major patients worldwide, causing significant morbidity (9). Studies have found that diabetes in thalassemia major patients is associated with pancreas volume, hypogonadism, hepatitis C infection, age, and ferritin levels (10). Iron overload is a key factor in diabetes in patients with thalassemia major, but the exact mechanism is still unclear, and it may be mediated by several key mechanisms, such as insulin deficiency and resistance, genetic factors, liver dysfunction, and autoimmunity in the absence of anti-islet cell antibodies. Researchers have traditionally believed that diabetes is directly caused by iron-induced damage to pancreatic beta cells, resulting in a decrease in insulin secretory capacity (11). In addition, an important mechanism of insulin resistance includes the possibility that iron excess directly causes resistance through hepatic dysfunction (10). Recently, another proposed etiology for glucose abnormalities has been that iron overload may trigger the autoimmune response against β cells through oxidative damage, leading to selective β cell destruction (12). On the other hand, diabetes is thought to be associated with an increased risk of several types of cancer, such as endometrial, liver, pancreatic, colorectal, and breast cancer (13). Some studies have shown that insulin therapy may contribute to the risk of pancreatic and colorectal cancer, which in turn may be attributed to the activation of insulin receptors on neoplastic cells (14).

**Figure 3:**
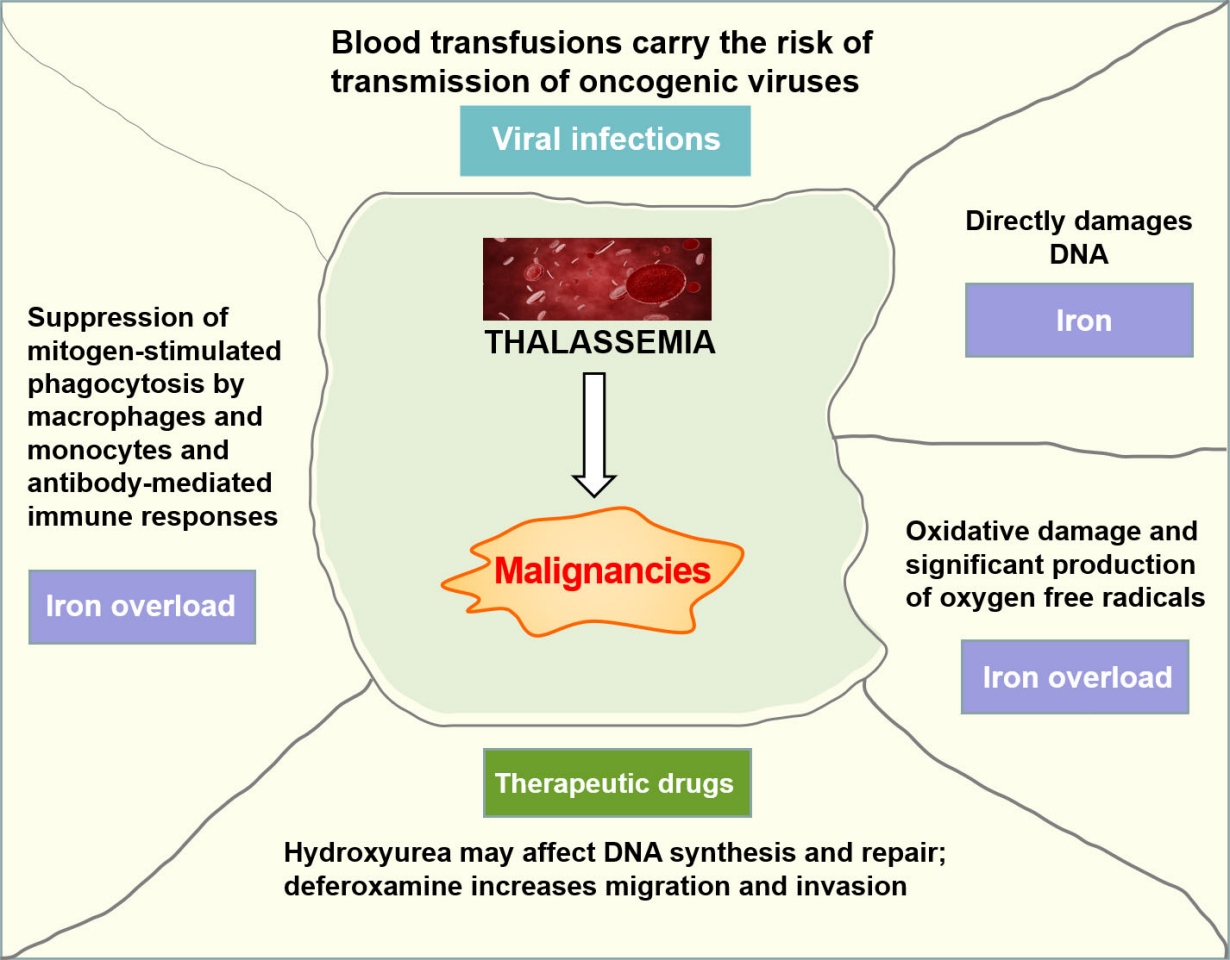
Possible mechanisms of solid malignancy development in thalassemia.

It has been suggested that patients with thalassemia seem to be predisposed to a higher risk of malignancy onset than the general population, especially at a young age. The patients with thalassemia and cancer had mainly hematologic malignancies, and a higher proportion of cancer was observed in patients with TDT than NTDT (15). Iron overload, transfusion-transmitted viruses, and immunosuppression from blood transfusions may all contribute to this increased risk (**Figure 3**). Iron has been shown to be carcinogenic because non-transferrin-bound iron directly damages the DNA, leading to the deactivation of tumor suppressor proteins such as p53, and severe iron overload could result in iron-induced oxidative damage and significant production of oxygen free radicals (16). Beyond that, iron overload may favor the growth of malignant cells through an imbalance in immune regulation caused by suppression of mitogen-stimulated phagocytosis by macrophages and monocytes, antibody-mediated immune responses, and modulation of cytokine activities and lymphocyte distribution in different regions of the immune system (17).

Blood transfusions carry the risk of transmitting oncogenic viruses that are responsible for at least some specific forms of cancer. The viruses most likely to be involved are Epstein–Barr virus (EBV), hepatitis virus, human herpes virus (HHV), and human T-lymphotropic virus 1 (HTLV1) (16). Previous studies have confirmed that EBV has an underlying relationship with the pathogenesis of nasopharyngeal carcinoma and lymphoma. HHV-8 is considered to be responsible for Kaposi’s sarcoma. HTLV-1 may be responsible for the development of adult T-cell leukemia/lymphoma, and the hepatitis virus has been correlated with hepatocellular carcinoma (HCC).

It is known that hydroxyurea can affect DNA synthesis and repair, resulting in mutations and subsequent chromosomal damage, and has been used as a treatment for thalassemia to reduce the need for transfusions (18). Although there is no evidence that hydroxyurea has increased carcinogenic potential in thalassemia patients, some researchers have raised many concerns about the carcinogenic potential of this drug. Recent data have emerged on the underlying role of iron chelation therapy in promoting cancer. Deferoxamine enhanced the migration and invasion of colon cancer cell lines *in vitro* through a process consistent with epithelial-mesenchymal metastasis and the metastatic potential of breast cancer cell lines through the hypoxia-inducible factor-1α pathway (19).

It should be noted that, as the primary treatment for nasopharyngeal cancer, radiation therapy is also a known risk factor for second primary cancer (SPC) (20). According to reports, the incidence of SPCs in patients with NPC after radiotherapy ranged from 0.04% to 7%, and the median latency ranged from 7.6 to 17.0 years (21). However, not all SPCs that occur after radiotherapy are due to irradiation, and in most cases, it is difficult to distinguish radiation-related SPCs from sporadic malignancies.

## Conclusion

In conclusion, malignancy and endocrinopathy are gradually emerging as threatening morbidities in patients with thalassemia. We hope that this case report will stimulate the development of a more sophisticated and forward-looking analysis of cancer and endocrine disorders in thalassemia and may help clinicians further understand this disease.

## Data availability statement

The original contributions presented in the study are included in the article/supplementary material. Further inquiries can be directed to the corresponding author.

## Ethics statement

Written informed consent was obtained from the individual(s) for the publication of any potentially identifiable images or data included in this article.

## Author contributions

XY and YP made diagnostic assessment and therapeutic intervention. TN conducted information collection and patient follow-up. WS made pathological diagnosis and analysis. YZ and YP conducted the literature review. XY drafted the manuscript. YZ revised the manuscript. All authors reviewed the manuscript. XY and YP contributed equally. All authors contributed to the article and approved the submitted version.

## References

[1] Vichinsky E. Complexity of alpha thalassemia: growing health problem with new approaches to screening, diagnosis, and therapy. Ann New York Acad Sci (2010) 1202:180–7. doi: 10.1111/j.1749-6632.2010.05572.x 20712791

[2] Weatherall DJ. The challenge of haemoglobinopathies in resource-poor countries. Br J Haematol (2011) 154(6):736–44. doi: 10.1111/j.1365-2141.2011.08742.x 21726207

[3] El-Beshlawy A El-Ghamrawy M. Recent trends in treatment of thalassemia. Blood Cells Mol Dis (2019) 76:53–8. doi: 10.1016/j.bcmd.2019.01.006 30792169

[4] Taher AT Cappellini MD. How I manage medical complications of β-thalassemia in adults. Blood (2018) 132(17):1781–91. doi: 10.1182/blood-2018-06-818187 30206117

[5] Taher AT Weatherall DJ Cappellini MD. Thalassaemia. Lancet (London England) (2018) 391(10116):155–67. doi: 10.1016/s0140-6736(17)31822-6 28774421

[6] Weatherall DJ. Thalassaemia: the long road from bedside to genome. Nat Rev Genet Aug (2004) 5(8):625–31. doi: 10.1038/nrg1406 15266345

[7] Borgna-Pignatti C Rugolotto S De Stefano P Zhao H Cappellini MD Del Vecchio GC. Survival and complications in patients with thalassemia major treated with transfusion and deferoxamine. Haematologica (2004) 89(10):1187–93. 15477202

[8] Chung WS Lin CL Lin CL Kao CH. Thalassaemia and risk of cancer: a population-based cohort study. J Epidemiol Community Health (2015) 69(11):1066–70. doi: 10.1136/jech-2014-205075 25922472

[9] Chatterjee R Bajoria R. New concept in natural history and management of diabetes mellitus in thalassemia major. Hemoglobin (2009) 33(Suppl 1):S127–30. doi: 10.3109/09553000903347880 20001615

[10] Li MJ Peng SS Lu MY Chang HH Yang YL Jou ST. Diabetes mellitus in patients with thalassemia major. Pediatr Blood Cancer Jan (2014) 61(1):20–4. doi: 10.1002/pbc.24754 24115521

[11] De Sanctis V Soliman AT Elsedfy H Pepe A Kattamis C El Kholy M. Diabetes and glucose metabolism in thalassemia major: an update. Expert Rev Hematol (2016) 9(4):401–8. doi: 10.1586/17474086.2016.1136209 26697756

[12] Monge L Pinach S Caramellino L Bertero MT Dall'omo A Carta Q. The possible role of autoimmunity in the pathogenesis of diabetes in B-thalassemia major. Diabetes Metab Apr (2001) 27(2 Pt 1):149–54. 11353881

[13] Kim DS Scherer PE. Obesity, diabetes, and increased cancer progression. Diabetes Metab J (2021) 45(6):799–812. doi: 10.4093/dmj.2021.0077 34847640 PMC8640143

[14] Leitner BP Siebel S Akingbesote ND Zhang X Perry RJ. Insulin and cancer: a tangled web. Biochem J (2022) 479(5):583–607. doi: 10.1042/BCJ20210134 35244142 PMC9022985

[15] Karimi M Giti R Haghpanah S Azarkeivan A Hoofar H Eslami M. Malignancies in patients with beta-thalassemia major and beta-thalassemia intermedia: a multicenter study in Iran. Pediatr Blood Cancer (2009) 53(6):1064–7. doi: 10.1002/pbc.22144 19533641

[16] Zanella S Garani MC Borgna-Pignatti C. Malignancies and thalassemia: a review of the literature. Ann New York Acad Sci (2016) 1368(1):140–8. doi: 10.1111/nyas.13005 26916208

[17] Huang X. Does iron have a role in breast cancer? Lancet Oncol (2008) 9(8):803–7. doi: 10.1016/s1470-2045(08)70200-6 PMC2577284 18672216

[18] Halawi R Cappellini MD Taher A. A higher prevalence of hematologic Malignancies in patients with thalassemia: Background and culprits. Am J Hematol (2017) 92(5):414–6. doi: 10.1002/ajh.24682 28195443

[19] Zhang W Wu Y Yan Q Ma F Shi X Zhao Y. Deferoxamine enhances cell migration and invasion through promotion of HIF-1α expression and epithelial-mesenchymal transition in colorectal cancer. Oncol Rep (2014) 31(1):111–6. doi: 10.3892/or.2013.2828 24173124

[20] Lee HF Lan JH Chao PJ Ting HM Chen HC Hsu HC. Radiation-induced secondary Malignancies for nasopharyngeal carcinoma: a pilot study of patients treated *via* IMRT or VMAT. Cancer Manag Res (2018) 10:131–41. doi: 10.2147/CMAR.S145713 PMC5783017 29403311

[21] Xi M Liu SL Zhao L Shen JX Zhang L Zhang P. Prognostic factors and survival in patients with radiation-related second Malignant neoplasms following radiotherapy for nasopharyngeal carcinoma. PloS One (2013) 8(12):e84586. doi: 10.1371/journal.pone.0084586 24367679 PMC3867505

